# Ethnobotanical study of plants used for traditional control of mosquitoes and other arthropod pests in the Ghibe valley, southwest Ethiopia

**DOI:** 10.1186/s41182-025-00740-6

**Published:** 2025-04-23

**Authors:** Zeyede Teshome, Alemtshay Teka, Abebe Animut, Mahlet Arage, Esayas Aklilu, Mirutse Giday

**Affiliations:** 1 Department of Biology, Woldia University, P.O. Box 50, Woldia, Ethiopia; 2 Aklilu Lemma Institute of Pathobiology, Addis Ababa University, P O Box 1176, Addis Ababa, Ethiopia

**Keywords:** Disease vectors, Ethnic groups, Indigenous knowledge, Insecticide, Malaria, Repellent

## Abstract

**Background:**

Medicinal plants have been used in the traditional healthcare system of Ethiopia, including controlling human biting mosquitoes. However, documented knowledge on such aspects remains scarce. In this study, plants used in the traditional control of mosquitoes and other arthropod vectors with the local knowledge and method of applications in the Ghibe valley of southwest Ethiopia were documented.

**Methods:**

Semi-structured interviews were used to collect ethnobotanical data between March and October 2024. A total of 361 informants consisting of 77 key informants and 284 general informants were selected using purposive and systematic random sampling methods, respectively, in Enor, Deri Saja Zuria, Misha and Sekoru districts of southwest Ethiopia. Frequency of citation and simple preference ranking were employed to determine the most used insecticidal and insect repellent plants. Relative importance of multipurpose plants was assessed using direct matrix ranking exercises. Independent samples t-test and one-way ANOVA tests were conducted to compare knowledge of informants on insecticidal and insect repellent plants.

**Results:**

A total of 53 plant species were used to control human biting insects. The most cited plant was *Allium sativum* L., (cited by 89%) followed by *Croton macrostachyus* Hochst. ex Delile (81%), *Olea europaea* subsp. *cuspidata* (Wall.G.Don) Cif. (77%), *Coleus abyssinicus* (Fresen.) A.J.Paton (69%; *n* = 361)*, Calpurnia aurea* (Aiton) Benth. (63%), *Juniperus procera* Hochst. ex Endl. (63%), *Echinops kebericho* Mesfin (58%), *Eucalyptus globulus* Labill (56%), *Melia azedarach* L. (52%) and *Phytolacca dodecandra* L'Hér. (36%). The results of the current study showed that different informant groups had considerably different level of knowledge on traditional usage of insecticidal and insect repellent plants.

**Conclusions:**

Plant species *Eucalyptus globulus*, *Calpurnia aurea*, *Phytolacca dodecandra*, *Echinops kebericho*, *Croton macrostachyus and Juniperus procera* were more frequently cited to be insecticides against human biting arthropods while *Melia azedarach* L., *Olea europaea* subsp. *cuspidata*, *Coleus abyssinicus, Croton macrostachyus, Eucalyptus globulus, Lippia abyssinica* (Otto & A. Dietr.) Cufod., and *Juniperus procera* were more frequently reported to be repellents. Some of these plants (*Coleus abyssinicus, Croton macrostachyus* and *Echinops kebericho*) have not yet been investigated in depth and thus require scientific evaluation for their efficacy as insecticides and or repellents against malaria-transmitting mosquitoes in Ethiopia.

## Background

Arthropods comprise diverse groups of species, which carry many disease-causing pathogens, parasitize humans and animals and are pests of human concerns [1–3]. Several hematophagous arthropods transmit human diseases [4]. Among these, mosquitoes transmit malaria, filariasis and arboviral diseases that cause severe morbidity, mortality and slower rate of economic development [5, 6]. Malaria alone caused about 249 million cases and 608 000 deaths in the year 2022 [7] and keeps 3.3 billion people at risk across 106 nations in the globe [8]. The leading vector of malaria in Ethiopia is *Anopheles arabiensis* [9] while *An. nili*, *An. pharoensis* and *An. funestus* serve as secondary vectors in limited foci [10].

Currently, the most essential malaria control strategy is vector control using chemical insecticides [11]. Long-lasting insecticide-treated mosquito nets (LLINs), indoor residual insecticide spraying (IRS) and mosquito larvae habitat modifications are the most popular control strategies of malaria vectors [12–15]. However, LLINs and IRS have limited effect on mosquitoes that are active during the early hours of the night and have exophagic and exophilic behaviors. Although insecticides are effective as insecticides and repellents, the emergence of insecticide resistant vectors and the poisoning effect of the insecticides on environment, humans and non-target organisms remains a challenge [16]. For instance, *An. arabiensis* has developed resistance to most of the available chemical insecticides [17]. This, together with the absence of effective antimalarial vaccine [18] and increasing evidences of *Plasmodium falciparum* resistance to coartem requires urgent need for new therapeutics [19]. To this end, plant-based insecticides and repellants can be effective, low cost, sustainable and environmental-friendly [20].

Humans have been using plants in their traditional healthcare system to control hematophagous arthropods that are medically and veterinary important since ancient times [21, 22]. Communities used selected plants to protect mosquitoes and the diseases they carry [21, 23]. Pyrethrum and neem are examples of plant products used to control mosquitoes [21, 24]. Consequently, attention has been given to plants as possible sources of vector control tools [25]. Various plants have been extracted and evaluated for their insecticidal and repellency properties against mosquitoes [25].

In Ethiopia, knowledge and practices about medicinal plants have been passed through generations in a hierarchical manner, and the majority of the plant species have limited ethnobotanical documentation [24, 26, 27]. This could lead to the loss or distortion of indigenous knowledge and the habit of using plants as insecticides and insect repellents in the country [27]. Thus, there is a need to preserve and protect the traditional knowledge on plants and evaluate their effectiveness against disease transmitting arthropods including mosquitoes [24, 28–30]. Knowledge and usage of plants as insecticides and insect repellents particularly for the control of malaria vectors was explored among the Yem ethnic group in Deri Saja Zuriya district, Gurage in Enor, Hadiya in Misha and Oromo in Sekoru, in the Ghibe valley, southwest Ethiopia.

## Study area and methods

### Description of the study districts

**Fig. 1:**
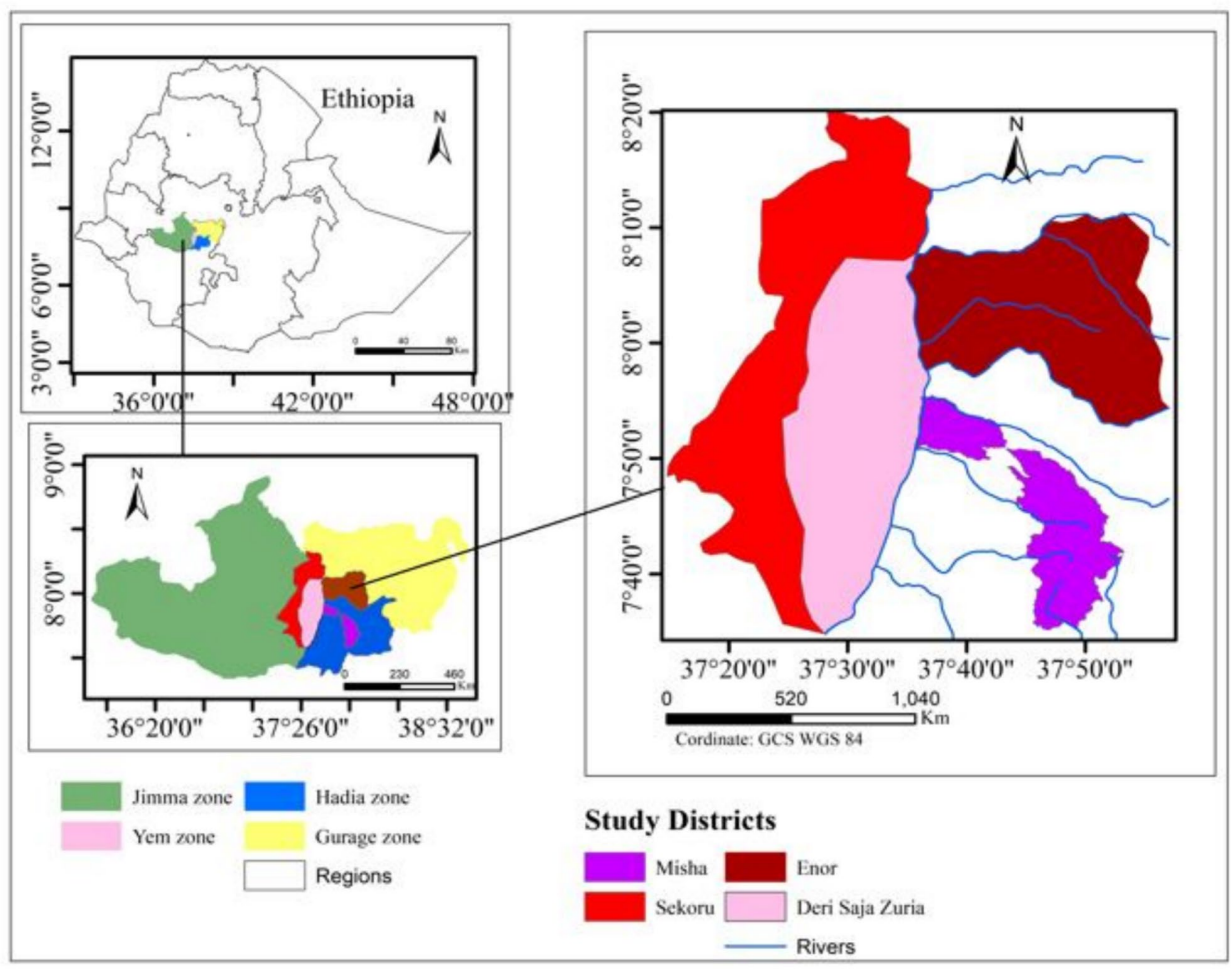
Map showing the geographical locations of study districts in Ghibe valley, southwest Ethiopia

The study was carried out in Deri Saja Zuria (in Yem zone), Enor (Gurage zone), Misha (Hadiya zone), and Sekoru (Jimma zone) districts, the Ghibe Valley, southwestern Ethiopia (Fig. 1).

#### Deri Saja Zuria district

The district is situated at 7° 58' N, 37° 26' E, and at 240 km away from Addis Ababa, the capital of Ethiopia. The majority of the inhabitants belong to the Yem people. The attitude of the lowland area is 817 m above sea level (m. a.s.l). while the highest peak is 2940 m above sea level (m.a.s.l) [31]. It receives a mean annual rainfall of 900–2200 mm in a bimodal pattern and temperature of 12 – 30 °C. The residents raise livestock and cultivate crops. The rain-fed agriculture activity primarily cultivates enset (*Ensete ventricosum*) followed by grains, and avocado (*Persea americana*) [31]. The crops consist of wheat (*Triticum aestivum*), barley (*Hordeum vulgare*), teff (*Eragrostis tef*), maize (*Zea mays*), sorghum (*Sorghum bicolor*), and pulses. According to archival malaria trend data and personal discussion with the local health extension worker in April 2024, the district is malarious in which the transmission occurs at the end of long rainy seasons in kiremt (mid-June to mid-September) and belg short rainy (February to May) seasons.

#### Enor district (Gurage zone)

Enor, 8°0′ N, 37°49′ E. It is at about 220 km away from Addis Ababa. The majority belong to the Gurage community. The elevation ranges between 800 and 3400 m.a.s.l. Rainfall is bimodal, with long rains occurring between June and September and a brief rainy season from February to May. The average annual temperature is 12.6–20.0 °C, and the average annual precipitation is between 1401 and 1800 mm. *Ensete ventricosum*, *Hordeum vulgare*, *Pisum sativum*, and *Phaseolus vulgaris*, *Eragrostis tef*, *Catha edulis*, and *Zea mays* are grown [32]. In the lowland areas malaria transmission occurs at the end of rainy seasons in kiremt (mid-June to mid-September) and belg (February to May).

#### Misha district (Hadiya zone)

Misha, 7°08’ N, 37°81’ E. It is located at about 250 km from Addis Ababa. Its altitude ranges between 500 and 3200 m a.s.l. The annual temperature ranges from 18–25 °C and the unimodal rainfall ranges from 1000–1500 mm. Animal rearing and crop production are practiced widely [33]. Fifty-four percent of the woreda lies in the range of Dega agro-climate (2400–3200 m a.s.l), 39% in the Woyna Dega (1800–2400 m a.s.l), and 7% in the Kola agro-climate (500–1800 m a.s.l) zones. The Woreda comprises undulating topography with vegetation of mild tropical rainforest type. The commonly cultivated crops in include *Hordeum vulgare*, *Pisum sativum*, and *Phaseolus vulgaris*, *Eragrostis tef*, *Catha edulis*, and *Zea mays*, coffee and vegetables. Malaria transmission occurs at the end of long and short rainy seasons in kiremt (mid-June to mid-September) and belg (February to May) [34].

#### Sekoru district (Jimma zone)

Sekoru is located between 7^o^ 35', 37° 15' E and the altitude rages between 1160 to 2940 m a.s.l. It is about 275 km away from Addis Ababa. High and medium-sized, rugged mountain ranges that expand into sloping plains and plateaus, gorges, and deeply carved river basins are all part of the topographic pattern. The region experiences hot, dry weather with an average yearly temperature of 19.2 °C and 1300–1800 mm rainfall. The community practices mixed farming, which includes raising cattle and cultivation of *Zea mays, Sorghum bicolor* and *Eragrostis tef.* Malaria is a significant public health concern, accounting for over 50% of clinical cases at the household level. Malaria transmission occurs at the end of long and short rainy seasons in kiremt (mid-June to mid-September) and belg (February to May) [35].

### Study design and sample size determination

The ethnobotanical study employed a community-based cross-sectional design. Four districts located along the Ghibe valley in the Central Regional State of Ethiopia were purposively selected with the help of the local administrations and healthcare offices on the basis of representing different ethnic groups and the fact that they are largely malarious. A district is a third level of political administration in Ethiopia following a region and zone. Two kebeles (the smallest political administration unit in Ethiopia) were selected from each district on the basis of knowledge pertaining to plant use in the control of mosquitoes and other arthropod pests with the assistance of the local administration offices. Accordingly, the selected kebeles were Guna and Dirbara from Misha district, Kelta and Natri from Sekoru, Ashe and Dicha Gote from Deri Saja Zuria, and Gonche Bete and Gerenbo from Enor. Following the selection of the kebeles, the sample size was determined according to the formula given below [75]:

$$n=\frac{N}{1+N({e)}^{2}},$$

where “n” is the intended sample size, “N” is the total number of households (2400) from eight kebeles of the study districts, “e” is the level of precision 5% (0.05), and 1 is the probability of event occurring. *n* = 2400/1 + 2400(0.05)^2^ = 343 (minimum sample size). A total of 361 informants were selected by increasing the minimum sample size for the non-response rate by 5% (*n* = 18) informants. According to their respective total number of households, each of the eight kebeles had an equal proportion of the total sample size, which was determined by the total number of households in Enor (520), Deri Saja Zuria (650), Sekoru (590), and Misha (640) districts. The kebeles were Gonche Bete (46; *N* = 305) and Gerenbo (32; *N* = 215) from Enor, Ashe (47; *N* = 310) and Dicha Gote (51; N = 340) from Deri Saja Zuria, Kelta (53; *N* = 350) and Natri (36; *N* = 240) from Sekoru, and Guna (41; *N* = 275) and Dirbara (55; *N* = 365) from Misha districts.

### Informant selection and ethnobotanical data collection

Purposive and systematic random sampling methods were used to identify knowledgeable traditional medicine practitioners and general informants, respectively, from each Kebele. Snowball sampling method including peer recommendations from community members, especially the elderly and knowledgeable locals, were taken into consideration in identifying traditional herbalists (males and females) who were included as key informants [36]. General informants were identified during random visits to residence areas.

Ethnobotanical data were collected between March and October 2024 through individual semi-structured interviews, focus group discussions, participant observation and field guided walks with informants. Name, age, gender, level of education, occupation, religion and ethnic background of each informant were recorded. In addition, the name of the plant used as an insecticide or insect repellent, the plant part used, the preparation method, the application technique, the name of targeted insect and the particular problem caused by each insect were recorded. Interviews were made in Guragegna language in Enor district, Yemgna in Deri Saja Zuria, Afan Oromo in Sekoru and Hadyagna in Misha. Data on medicinal plant marketability were gathered by visiting nearby marketplaces. Independent walks were made with individual informants to facilitate in depth discussion and identification and gathering of the plants from their natural habitats. One focus group discussion was held per kebele in order to get further data on community-level plant knowledge and to cross-check data gathered through interviews [37]. Samples of the plants were collected with the help of informants and local field assistants. Taxonomic identification of plants was performed by botanist at the National Herbarium, Addis Ababa University (AAU), and voucher specimens were deposited at Aklilu Lemma Institute of Pathobiology, AAU.

### Data analysis

Data were entered, cleaned, analyzed and summarized using Microsoft Excel computer software. The following indexes were employed in data analyses. Frequency of citation (FC) for each mentioned plant species was determined by counting the number of informants who reported the use of the particular plant species [24].

Simple preference ranking was performed to identify the most preferred plant(s) used as insecticide and insect repellent in a community [37]. The highest rank went to the most preferred plant and the lowest rank to the least preferred. It was made by involving ten informants randomly selected from among the key informants who participated during individual interviews. They were requested one after another to rank six plants used as insecticides and another six plants used as insect repellents after given the plant species to arrange the plants based on their personal perception. Based on the total score of each species, the rank was determined. Furthermore, simple preference ranking exercises were also conducted among six selected and most scarce reported plants from each study district to determine plants claimed to be the scarcest by the community and to determine the highly important threatening factors of plants in the study areas.

Direct matrix ranking exercise was done in each district to measure the relative importance of multipurpose plants based on their different uses in addition to their medicinal uses by a local community [37]. The obtained value determined the probable pressure exerted on each plant by the local people. Based on information gathered from individual interviews, plant species were selected and their use diversity were listed for selected key informants. Each informant was then asked to assign use values (5 = best, 4 = very good, 3 = good, 2 = less used, 1 = least used, and 0 = not used). Accordingly, each value was summed up and ranked.

Jaccard’s similarity index [38] was determined to shows the extent of overlap in the use of insecticidal and insect repellent plants between four of the study districts following the formula:

$$\text{JI}=\frac{\text{c}}{(\text{a}+\text{b}+\text{c})},$$

where JI is Jaccard’s similarity index, a is number of species found only in habitat a, b is number of species found only in habitat b, and c is number of species common to both habitats a and b.

Independent samples t-test and one-way ANOVA tests were conducted using SPSS statistical software version 25 to compare knowledge of informants on insecticidal and insect repellent plants. Independent samples t-test was conducted between gender (male and female), experience (knowledgeable and ordinary) and education level (those who could not read and write and those who able to read and write). One-way ANOVA test was conducted among four study districts and among age groups (younger, middle and older age). The Duncan analysis method was also used for pairwise comparison (compares the means of variables to each other) between study districts and age groups. All statistical tests were considered to be significant at *p* < 0.05.

## Results

### Socio-demographic background of informants

The ages of the informants ranged from 22 to 88 years (Table 1). The majority (97.8%) were farmers, (77.3%) were able to read and write but the remaining (22.7%) could not read and write.

**Table 1:** Socio-demographic background of informants on use of plants to control biting insects, in Ghibe valley, southwestern Ethiopia, 2024

Variable	Enor (*n* = 78)	Misha (*n* = 96)	Sekoru (*n* = 89)	Deri Saja Zuria (*n* = 98)	Total (*n* = 361)
Gender					
Male	78.2%	60.4%	71.9%	71.4%	70%
Female	21.8%	39.6%	28.1%	28.6%	30%
Age					
22–39	28.2%	32.3%	37.1%	27.6%	31.3%
40 – 60	32.1%	33.3%	43.8%	41.8%	38%
> 60	39.8%	34.4%	19.1%	30.6%	30.8%
Educational status					
Couldn’t read and write	30.8%	29.2%	21.4%	11.2%	22.7%
Could read and write only	43.6%	21.9%	53.4%	51%	43.5%
Primary school	20.5%	31.3%	18%	33.7%	26.3%
Secondary school	5%	13.5%	–	9.2%	5.3%
Higher education	–	4.2%	2.3%	2%	2%
Experience					
Key/knowledgeable	28.2%	14.6%	20.2%	23.5%	21%
General informant	71.8%	85.4%	79.8%	76.5%	79%
Total	21.6%	26.6%	24.7%	27.2%	100%

### Plant taxa being used to control insects

The plant species most frequently used in the traditional control of biting arthropods was *Allium sativum* L. (cited by 321 informants) followed by *Croton macrostachyus* Hochst.ex Delile (293)*, Olea europaea* subsp. *cuspidata* (Wall. G. Don) Cif. (279), *Coleus abyssinicus* (Fresen.) A. J. Paton (249), *Juniperus procera* Hochst. ex Endl. (227), *Calpurnia* a*urea* Aiton) Benth. (227)*, Echinops kebericho* Mesfin (209)*, Eucalyptus globulus* Labill (203)*, Carica papaya* L (197), *Ajuga integrifolia* Buch. -Ham. ex D. Don (194) and *Melia azedarach* L. (189) (Table 2).

**Table 2:** Plants used for traditional control of insects with frequency of citation (FC) in Ghibe valley, southwestern Ethiopia, 2024

Plant species	Districts									
	Enor (*n* = 78)		Deri Saja Zuria (*n* = 98)		Sekoru (*n* = 89)		Misha (*n* = 96)		Total (*n* = 361)	
	FC	%	FC	%	FC	%	FC	%	FC	%
*Ajuga integrifolia*	54	69	71	72	69	74	0	0	194	54
*Allium sativum*	74	75	92	94	79	84	76	80	321	89
*Aloe* sp.	17	22	0	0	0	0	0	0	17	4.7
*Artemisia annua*	0	0	32	32	0	0	0	0	32	8.8
*Brucea antidysenterica*	0	0	0	0	0	0	38	39	38	11
*Calpurnia aurea*	0	0	76	78	88	92	83	86	227	63
*Capsicum frutescens*	14	18	0	0	0	0	0	0	14	4
*Carica papaya*	38	49	83	84	76	76	0	0	197	55
*Carissa spinarum*	0	0	0	0	12	12	0	0	12	3.3
*Citrus medica*	13	17	0	0	0	0	0	0	13	3.6
*Clausena anisata*	0	0	0	0	0	0	17	18	17	5
*Clutia abyssinica*	0	0	0	0	7	7	0	0	7	1.9
*Coleus abyssinicus*	18	23	81	83	74	83	76	0	249	69
*Coleus maculosus* subsp. *edulis*	37	47	0	0	0	0	0	0	37	10
*Commelina* sp.	0	0	6	6	0	0	0	0	6	1.7
*Croton macrostachyus*	54	69	72	73	65	69	82	86	293	81
*Cucumis dipsaceus*	7	9	0	0	0	0	0	0	7	2
*Dicliptera laxata*	0	0	1	1	0	0	0	0	1	0.2
*Echinops kebericho*	32	41	74	75	68	68	35	36	209	58
*Embelia schimperi*	17	22	0	0	0	0	0	0	17	4.7
*Eucalyptus globulus*	63	71	37	38	31	35	72	75	203	56
*Euphorbia tirucalli*	0	0	13	13	0	0	0	0	13	3.6
*Foeniculum vulgare*	23	29	0	0	0	0	0	0	23	6
*Fuerstia africana*	3	4	0	0	0	0	0	0	3	0.8
*Gardenia ternifolia*	6	8	0	0	0	0	0	0	6	1.7
*Hypericum quartinianum*	0	0	0	0	3	3	0	0	3	0.8
*Impatiens* sp.	0	0	0	0	27	27	0	0	27	7.5
*Juniperus procera*	71	61	38	38	44	46	74	77	227	63
*Justicia schimperiana*	52	67	0	0	0	0	0	0	52	14
*Lagenaria siceraria*	0	0	66	67	37	37	0	0	103	29
*Leucas calostachys*	11	14	0	0	0	0	0	0	11	3
*Lippia abyssinica*	0	0	29	30	58	62	0	0	87	22
*Maesa lanceolata*	0	0	0	0	1	1	0	0	1	0.2
*Melia azedarach*	14	18	87	88	60	67	28	29	189	52
*Nicotiana tabacum*	0	0	61	62	58	58	0	0	119	33
*Nigella sativa*	0	0	11	11	51	51	0	0	62	17
*Ocimum basilicum*	11	14	0	0	0	0	0	0	11	3
*Ocimum gratissimum*	0	0	57	58	0	0	0	0	57	16
*Olea europaea* subsp. *cuspidata*	61	78	72	73	79	89	67	70	279	77
*Pentanema confertiflorum*	0	0	1	1	0	0	0	0	1	0.2
*Persea americana*	32	41	0	0	0	0	0	0	32	8.8
*Phytolacca dodecandra*	0	0	79	80	0	0	52	53	131	36
*Premna schimperi*	0	0	16	16	35	39	0	0	51	14
*Protea gaguedi*	0	0	3	3	0	0	0	0	3	26
*Rhamnus prinoides*	0	0	3	3	0	0	0	0	3	0.8
*Rosa abyssinica*	0	0	0	0	4	4	0	0	4	1.1
*Ruta chalepensis*	57	73	44	45	0	0	0	0	101	24
*Senna septemtrionalis*	0	0	51	52	0	0	0	0	51	16
*Sida rhombifolia*	0	0	3	3	0	0	0	0	3	0.8
*Solanecio mannii*	0	0	0	0	1	1	0	0	1	0.2
*Tetradenia* sp.	0	0	1	1	0	0	0	0	1	0.2
*Thymus schimperi*	0	0	1	1	0	0	0	0	1	0.2
*Trigonella foenum-graecum*	0	0	0	0	48	48	0	0	48	13

A total of 53 plant species belonging to 31 families and 53 genera were being used by the people residing along the Ghibe valley in southwestern Ethiopia to control mosquitoes and other arthropod pests. The most widely used families were Lamiaceae (10 species), Asteraceae (4 species), Fabaceae (3 species), and Rutaceae (3 species). Majority (66%; *n* = 53) were used as insect repellents and the others (34%; *n* = 53) as both insecticides and insect repellents. The target insects reported by 96.6% of the informants were mosquitoes. The most frequently used plant part is the leaf (53%) followed by the shoot (16%), root (9%), seed (7%), bark (5), stem (2), bulb (2) and flower (2). The most frequently used mode of applications either to kill or repel insects is drinking juice (60%) followed by house fumigation (28%), bathing the body with smoke and vapor (13%), spraying powder of dry leaf (8%), pasting the juice on the body (7.5) and direct placing of fresh leaf (7.5%) (Table 3).

**Table 3:** List of plants traditionally claimed to have insecticidal and repellent properties in Ghibe valley, southwest Ethiopia, 2024

Scientific name, family name, voucher number	Local names	Habitat	Habit	Claimed use	Target insect	Parts used and method of application	Other medicinal uses	Districts
*Ajuga integrifolia,* Buch. -Ham. ex D. Don, Lamiaceae, Z-04-2024	Armagusa	Wild	Herb	Repellent	Mosquito	Drink fresh leaves and stem juice & cover oneself with vapor	Anti-malarial	Enor, Deri Saja Zuria & Sekoru
*Allium sativum* L., Alliaceae, Z-07-2024	Nech Shinkurt	Cultivate	Herb	Insecticide & Repellent	Mosquito	Eating & fumigate with vapor & paste bulb juice on skin	Anti-malarial, common cold	Enor, Deri Saja Zuria, Misha & Sekoru
*Aloe* sp., Aloaceae*,* Z-08-2024	Eret	Wild	Herb	Repellent	Mosquito	Drink juice of fresh leaves & paste on skin	Anti-malarial	Enor
*Artemisia annua* L.., Asteraceae, Z-43-2024	Arity	Home garden	Herb	Repellent	Mosquito	Chewing leaves	Anti-malarial	Deri Saja Zuria
*Brucea antidysenterica* J.F.Mill., Simaroubaceae, Z-46-2024	Chironta	Wild	Shrub	Repellent	Mosquito Any insect	Direct placing of fresh leaves & chewing freshly	Anti-malarial	Misha
*Calpurnia aurea* (Aiton) Benth., Fabaceae, Z-30-2024	Cheka	Wild	Shrub	Insecticide & Repellent	Any insect Mosquito Weevil	Direct placing of fresh leaf & spraying powder		Deri Saja Zuria, Misha & Sekoru
*Capsicum frutescens* L., Solanaceae, Z-17-2024	Mitmita	Cultivate	Herb	Insecticide & Repellent	Weevil	Spraying with fruit powder		Enor
*Carica papaya* L., Caricaceae, Z-38-2024	Papaya	Cultivate	Tree	Repellent	Mosquito	Drink whole plant juice	Anti-malarial	Enor, Deri Saja Zuria & Sekoru
*Carissa spinarum* L., Apocynaceae, Z-51-2024	Agamsa	Wild	Tree	Repellent	Mosquito	Drink leaves juice	Anti-malarial	Sekoru
*Citrus medica* L.*,* Rutaceae, Z-02-2024	Tiringo	Cultivate	Tree	Repellent	Mosquito	Swallow leaves juice	Anti-malarial	Enor
*Clausena anisata* (Willd.) Hook.f. ex Benth.Rutaceae, Z-57-2024	Baytehaqa	Wild	Shrub	Repellent	Mosquito	drink leaves juice	Anti-malaria, other illness	Misha
*Clutia abyssinica* Jaub. & Spach, Euphorbiaceae, Z-49-2024	Nagina	Wild	Tree	Repellent	Mosquito	Drink leaves juice	Anti-malarial	Sekoru
*Coleus abyssinicus* (Fresen.) A.J.Paton. Lamiaceae, Z-10–2024	Fuanfua	Home garden	Shrub	Insecticide & Repellent	Mosquito, fly	Drink leaves juice, fumigate house with smoke & vapor & direct placing of fresh leaf	Anti-malarial, common cold, eye illnesses	Enor, Deri Saja Zuria, Misha & Sekoru
*Coleus maculosus* subsp. *edulis* (Vatke) A.J.Paton, Lamiaceae, Z-20-2024	Yegurage Dinich	Wild	Herb	Repellent	Mosquito	Drink leaves juice	Anti-malarial	Enor
*Commelina* sp., Commelinaceae, Z-28-2024	Laluncha	Wild	Herb	Repellent	Mosquito	Drink leaves juice	Anti-malarial	Deri Saja Zuria
*Croton macrostachyus* Hochst. ex Delile*,* Euphorbiaceae, Z-13-2024	Wanshehna	Wild	Tree	Insecticide & Repellent	Mosquito, house fly	House fumigation with smoke of fresh plants Drinking leaves juice	Anti-malarial	Enor, Deri Saja Zuria, Misha & Sekoru
*Cucumis dipsaceus* L.f.*,* Cucurbitaceae, Z-15-2024	Afer Tay	Wild	Herb	Repellent	Mosquito	Drink root juice	Anti-malarial, other illnesses	Enor
*Dicliptera laxata* C.B.Clarke, Acanthaceae, Z-42-2024	Togo	Wild	Herb	Repellent	Mosquito	Drink leaves juice	Anti-malarial	Deri Saja Zuria
*Echinops kebericho* Mesfin., Asteraceae, Z-37-2024	Quebracho	Cultivate	Herb	Insecticide & Repellent	Mosquito house fly	House fumigation with root smoke		Enor, Deri Saja Zuria, Misha & Sekoru
*Embelia schimperi* Vatke.*,* Lamiaceae, Z-16-2024	Koso	Wild	Tree	Repellent	Mosquito	Drink flower juice	Anti-malarial, parasitic illness	Enor, Deri Saja Zuria, Misha & Sekoru
*Eucalyptus globulus Labill,* Myrtaceae, Z-03-2024	Bahir Zaf	Home garden	Tree	Insecticide & Repellent	Mosquito, house fly, ants	Surround with fresh leaves, fumigate with vapor & bathe in smoke	Common cold	Enor, Deri Saja Zuria & Sekoru
*Euphorbia tirucalli* L.*,* Euphorbiaceae, Z-24-2024	Kinchib	Wild	Shrub	Insecticide & Repellent	Mosquito, house fly	Whole plant smoking, drink juice	Anti-malarial	Deri Saja Zuria
*Foeniculum vulgare* Mill., Apiaceae, Z-12-2024	Ensilal	Wild	Herb	Repellent	Mosquito	Drink juice of fresh leaves and stem	Anti-malarial	Enor
*Fuerstia africana* T.C.E.Fr. Lamiaceae, Z-18-2024	Kiskas	Wild	Herb	Repellent	Mosquito	Drink whole plant juice	Anti-malarial	Enor
*Gardenia ternifolia* Schumach. & Thonn.*,* Rubiaceae, Z-06-2024	Gambelo	Home garden	Shrub	Repellent	Mosquito	Chewing bark and root	Anti-malarial	Enor
*Hypericum quartinianum* A.Rich., Hypericaceae, Z-52-2024	Fenchesis	Wild	Shrub	Repellent	Mosquito	Drink leaves juice	Anti-malarial	Sekoru
*Impatiens ethiopica* Grey-Wilson, Balsaminaceae, Z-58–2024	Enso Sila	Home garden	Herb	Repellent	Mosquito	Drink root juice	Anti-malarial	Sekoru
*Juniperus procera* (Hochst. ex Nees) T.Anderson, Cupressaceae, Z-09-2024	Tsid	Wild	Tree	Insecticide & Repellent	Mosquito, house fly	House fumigation with whole plant freshly and rarely dried		Enor, Deri Saja Zuria, Misha & Sekoru
*Justicia schimperiana* (Hochst. ex Nees) T.Anderson*,* Acanthaceae, Z- 01-2024	Hemba	Wild	Shrub	Repellent	Mosquito	Swallow leaves juice	Anti-malarial, stomach pain	Enor
*Lagenaria* siceraria (Molina) Standl., Cucurbitaceae, Z-39-2024	Bookie	Cultivate	Climber	Repellent	Mosquito	Drink leaves juice	Anti-malarial	Deri Saja Zuria, & Sekoru
*Leucas calostachys* Oliv.*,* Lamiaceae, Z-14-2024	Kiza	Wild	Herb	Repellent	Mosquito	Drink leaves juice	Anti-malarial	Enor
*Lippia abyssinica* (Otto & A.Dietr.) Cufod., Verbenaceae, Z-27-2024	Kussaye	Wild	Shrub	Insecticide & Repellent	Mosquito, house fly	Leaves and stem smoking		Deri Saja Zuria, & Sekoru
*Maesa lanceolata* Forssk*.*, Myrsinaceae, Z-53-2024	Ajebaye	Wild	Tree	Repellent	Mosquito	Drink leaves juice	Anti-malarial	Sekoru
*Melia azedarach* L., Meliaceae, Z-11-2024	Zayka	Home garden	Tree	Insecticide & Repellent	Mosquito, house fly	Smoke & sprayed house by powder of leaves, direct placing of leaves	Anti-malarial	Enor, Deri Saja Zuria, Misha & Sekoru
*Nicotiana tabacum* L. Solanaceae, Z-40-2024	Tinbaho	Wild	Herb	Insecticide & Repellent	Any insects	Smoking & sprayed house with leaves powder		Deri Saja Zuria, & Sekoru
*Nigella sativa* L., Ranunculaceae, Z-56-2024	Tikur Azmud	Cultivate	Herb	Repellent	Mosquito	Drink leaves juice	Anti-malarial	Deri Saja Zuria & Sekoru
*Ocimum basilicum* L., Lamiaceae, Z-19-2024	Beso Bila	Cultivate	Herb	Repellent	Mosquito	Drink leaves juice	Anti-malarial	Enor
*Ocimum gratissimum* L., Lamiaceae, Z-35-2024	Anichebe	Wild	Shrub	Insecticide & Repellent	Mosquito	Fumigate with vapor of boiled leaves		Deri Saja Zuria
*Olea europaea* subsp. cuspidata (Wall.G.Don) Cif., Oleaceae, Z-45-2024	Weira	Wild	Tree	Insecticide & Repellent	Mosquito, house fly	House fumigation with whole plant freshly and rarely dried		Enor, Deri Saja Zuria, Misha & Sekoru
*Pentanema confertiflorum* (A.Rich.) D.Gut.Larr., Asteraceae, Z-25-2024	Oyazo	Wild	Climber	Repellent	Mosquito	drink root juice	Anti-malarial, other diseases	Deri Saja Zuria
*Persea americana* Mill., Lauraceae, Z-21-2024	Avocado	Cultivate	Tree	Repellent	Mosquito	Drink root juice	Anti-malarial	Enor
*Phytolacca dodecandra* L'Hér.*, Phytolaccaceae,* Z-32-2024	Endod	Wild	Shrub	Insecticide & Repellent	Mosquito, lice, flies, flea, tick	Leaves smoke, spraying juice & washing body		Deri Saja Zuria, & Sekoru
*Premna schimperi* Engl., Lamiaceae, Z-36-2024	Urgessa	Wild	Shrub	Insecticide & Repellent	Mosquito	Fumigate with vapor of boiled leaves		Deri Saja Zuria, & Sekoru
*Protea gaguedi* J.F.Gmel.*,* Proteaceae, Z-23-2024	Korasuma	Wild	Shrub	Insecticide & Repellent	Mosquito, house fly	Stem smoking		Deri Saja Zuria, & Sekoru
*Rhamnus prinoides* L'Hér., Rhamnaceae, Z-22-2024	Gesho	Cultivate	Shrub	Insecticide & Repellent	Mosquito, lice	Drink leaves & fruit juice & paste on the body	Anti-malarial, anti-fungi	Deri Saja Zuria
*Rosa abyssinica* R.Br. ex Lindl., Rosaceae, Z-50-2024	Ganona	Wild	Shrub	Repellent	Mosquito	Drink leaves juice	Anti-malarial	Sekoru
*Ruta chalepensis* L.*,* Rutaceae, Z-05-2024	Tena Adam	Home garden	Herb	Insecticide & Repellent	Mosquito	Fumigate & bathing with vapor	Anti-malarial, common cold	Deri Saja Zuria & Enor
*Senna petersiana* (Bolle) Lock*,* Fabaceae, Z-31-2024	Semameki	Wild	Shrub	Repellent	Mosquito	house fumigation with leaves & stem smoke		Deri Saja Zuria
*Sida rhombifolia* L., Malvaceae, Z-26-2024	Karaba	Wild	Herb	Repellent	Mosquito	drink leaves juice	Anti-malarial	Deri Saja Zuria
*Solanecio mannii* (Hook.f.) C.Jeffrey, Asteraceae, Z-55-2024	Umiru	Home garden	Tree	Repellent	Mosquito	Drink leaves juice	Anti-malarial	Sekoru
*Tetradenia* sp., Lamiaceae, Z-34-2024	Banajo	Wild	Shrub	Repellent	Mosquito	Drink leaves juice	Anti-malarial	Deri Saja Zuria
*Thymus schimperi* Ronniger, Lamiaceae, Z-41-2024	Gitwa	Wild	Climber	Repellent	Mosquito	Drink leaves juice	Anti-malarial	Deri Saja Zuria
*Trigonella foenum-graecum* L., Fabaceae, Z-57-2024	Abish	Cultivate	Herb	Repellent	Mosquito	Drink leaves juice	Anti-malarial	Sekoru

#### Habitats and habits of plants used to control insects

**Fig. 2:**
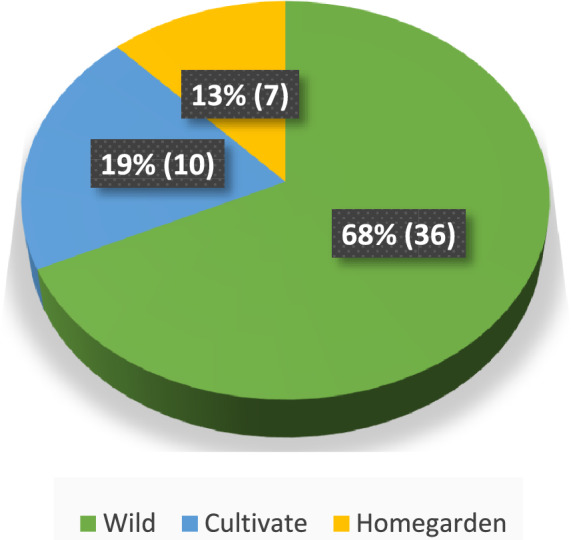
Habitats of the insecticidal and repellent plants in Ghibe valley, southwestern Ethiopia, 2024

**Fig. 3:**
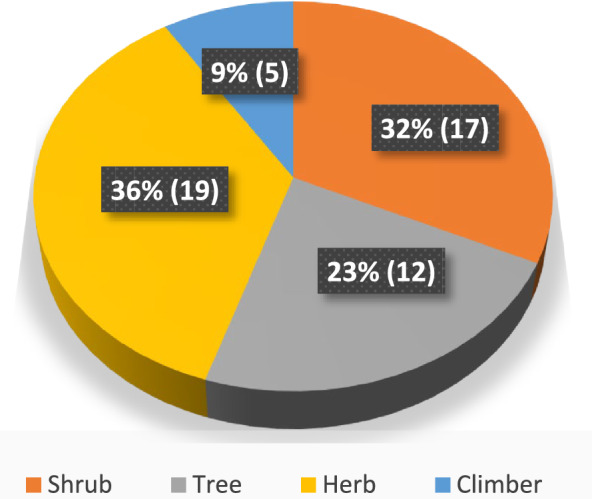
Habits of the insecticidal and insect repellent plants in Ghibe valley, southwestern Ethiopia, 2024

Most (68%; *n* = 53) of the reported insecticidal and repellent plants were uncultivated growing in the wild/semiwild habitats and few (13%; *n* = 53) were found in homegardens (Fig. 2). Most plants (36%; *n* = 53) were herbs, followed by shrubs (32%; *n* = 53) (Fig. 3).

### Preference ranking of plants

#### Preference ranking of selected insecticidal plants

Preference ranking values of the six frequently cited plants species used as insecticides in each study district are given in Table 4. In Deri Saja Zuria district, *P. dodecandra* was ranked as the first best insecticidal plant (49 scores) followed by *E. kebericho* (39). In Enor district, *E. globulus* was ranked as the most effective insecticidal plant (47) followed by *C. macrostachyus* that ranked as the second (44) most effective insecticidal plant. Similarly, *E. globulus* was ranked the first (49) preferred insecticidal plant followed by *J. procera* (45) in Misha district; whereas, *C. aurea* was ranked as first insecticidal plant (53) followed by *L. abyssinica* (47) in Sekoru district.

**Table 4:** Preference ranking of plants used as insecticides in Ghibe valley, southwestern Ethiopia, 2024

Scores given by key informants (A–K)												
Plant species	A	B	C	D	F	G	H	I	J	K	Total	Rank
Deri Saja Zuria												
*Melia azedarach*	2	2	1	3	6	2	2	6	6	6	36	3rd
*Echinops kebericho*	4	3	5	2	4	3	6	5	3	4	39	2nd
*Phytolacca dodecandra*	5	5	6	5	5	6	4	3	5	5	49	1st
*Protea gaguedi*	6	1	2	1	2	1	1	2	1	1	30	6th
*Nicotiana tabacum*	3	4	3	4	3	5	3	4	4	2	35	4th
*Calpurnia aurea*	1	6	4	6	1	4	5	1	2	3	33	5th
Enor												
*Olea europaea* subsp. *cuspidata*	2	6	2	2	6	2	6	1	1	2	30	4th
*Coleus abyssinicus*	3	3	4	6	3	5	3	3	6	3	39	3rd
*Eucalyptus globulus*	5	5	3	4	4	6	5	4	5	6	47	1st
*Croton macrostachyus*	4	4	5	5	5	4	4	5	4	4	44	2nd
*Juniperus procera*	6	1	6	1	2	1	2	6	2	1	28	5th
*Melia azedarach*	1	2	1	3	1	3	1	2	3	5	22	6th
Misha												
*Croton macrostachyus*	6	2	2	2	4	2	6	3	3	3	33	4th
*Eucalyptus globulus*	4	5	5	5	5	5	5	6	4	5	49	1st
*Olea europaea* subsp. *cuspidata*	3	6	6	3	2	6	3	2	6	1	38	3rd
*Brucea antidysenterica*	1	1	1	1	6	4	1	1	2	2	20	6th
*Juniperus procera*	5	4	4	6	3	3	4	5	5	6	45	2nd
*Coleus abyssinicus*	2	3	3	4	1	1	2	4	1	4	25	5th
Sekoru												
*Olea europaea* subsp. *cuspidata*	4	3	6	1	4	4	3	2	3	4	34	3rd
*Calpurnia aurea*	6	6	5	6	6	6	1	6	5	6	53	1st
*Echinops kebericho*	3	2	2	2	2	2	4	4	6	2	29	4th
*Lippia abyssinica*	5	5	4	5	3	5	6	5	4	5	47	2nd
*Premna schimperi*	1	1	1	4	5	3	2	3	1	1	22	6th
*Brucea antidysenterica*	2	4	3	3	1	1	5	1	2	3	25	5th

#### Preference ranking of selected insect repellent plants

In Deri Saja Zuria district, *M. azedarach* (52) was ranked as first highly effective insect repellent plant followed by *C. abyssinicus* (47) that ranked as the second highly effective insect repellent plant. In Enor district, *E. globulus* (50) was ranked as first most effective insect repellent plant followed by *C. macrostachyus* (44) that ranked as the second most effective insect repellent plant (Table 5).

**Table 5:** Preference ranking of plants used as insect repellents in Ghibe valley, southwestern Ethiopia, 2024

Scores given by key informants (A–K)												
Plant species	A	B	C	D	F	G	H	I	J	K	Total	Rank
Deri Saja Zuria												
*Melia azedarach*	5	6	6	5	6	4	4	6	5	5	52	1st
*Coleus abyssinicus*	4	3	5	6	4	6	5	5	3	6	47	2nd
*Calpurnia aurea*	6	2	2	3	1	2	6	1	2	3	28	4th
*Ocimum gratissimum*	1	1	1	1	2	1	1	2	6	1	17	6th
*Senna septemtrionalis*	3	4	3	4	3	5	3	4	4	2	35	3rd
*Echinops kebericho*	2	5	4	2	5	3	2	3	1	4	31	5th
Enor												
*Allium sativum*	2	2	2	2	6	2	1	1	6	2	26	5th
*Coleus abyssinicus*	3	3	4	3	3	5	3	6	3	6	39	3rd
*Eucalyptus globulus*	5	6	6	6	4	3	6	4	5	5	50	1st
*Croton macrostachyus*	4	4	5	5	5	4	4	5	4	4	44	2nd
*Ajuga integrifolia*	1	1	1	1	2	1	2	2	2	1	14	6th
*Ruta chalepensis*	6	5	3	4	1	6	5	3	1	3	37	4th
Misha												
*Croton macrostachyus*	2	2	2	6	6	2	2	3	6	3	34	4th
*Eucalyptus globulus*	4	5	5	5	5	6	5	4	4	5	48	1st
*Olea europaea* subsp. *cuspidata*	6	6	6	3	2	5	3	6	1	6	44	2nd
*Clausena anisata*	1	1	1	1	4	4	1	1	2	2	18	6th
*Juniperus procera*	5	4	4	4	3	3	6	5	5	4	43	3rd
*Coleus abyssinicus*	3	3	3	2	1	1	4	2	3	1	23	5th
Sekoru												
*Olea europaea* subsp. *cuspidata*	6	5	6	6	4	6	3	5	6	6	53	1st
*Calpurnia aurea*	2	4	5	3	6	1	6	1	5	3	36	3rd
*Echinops kebericho*	3	6	2	2	2	2	4	4	2	2	29	4th
*Coleus abyssinicus*	5	3	4	5	3	5	5	6	4	5	45	2nd
*Premna schimperi*	1	1	1	4	5	3	2	3	1	1	22	6th
*Lippia abyssinica*	4	2	3	1	1	4	1	2	3	4	24	5th

Similarly, *E. globulus* (48) was ranked as first greatest insect repellent plant followed by *O. europaea* subsp. *cuspidata* (44) that ranked as the second greatest insect repellent plant in Misha district; whereas, *O. europaea* subsp. *cuspidata* (53) was ranked as first best insect repellent plant followed by *C. abyssinicus* (45) that were ranked second-best insect repellent plants in Sekoru district (Table 5)

#### Preference ranking of selected plants used in insect control based on level of scarcity

Preference ranking values of six selected insecticidal and insect repellent plants from each study district based on their degree of scarceness are given in Table 6. In Deri Saja Zuria district, *E. schimperi* is most scarce plant (51) followed by *P. gaguedi* (47) while *O. europaea* subsp. *cuspidata* (15) is the least scarce. In Enor district, *F. africana* is most scarce plant (49) followed by *C. dipsaceus* (46) while *M. azedarach* (21) is the least scarce. Similarly, *B. antidysenterica* is most scarce plant (44) followed by *C. anisata* (42) while *C. macrostachyus* (27) is the least scarce plant in Misha district; whereas, *S. mannii* is most scarce plant (48) followed by *E. schimperi* (43) while *H. quartinianum* (17) is least scarce plant in Sekoru district.

**Table 6:** Preference ranking of insecticidal and repellent plants based on scarceness in Ghibe valley, southwestern Ethiopia, 2024

Scores given by key informants (A–K)												
Plant species	A	B	C	D	F	G	H	I	J	K	Total	Rank
Deri Saja Zuria												
*Protea gaguedi*	3	4	6	5	5	4	3	6	6	5	47	2nd
*Phytolacca dodecandra*	4	3	5	4	4	6	5	3	4	2	40	3rd
*Thymus schimperi*	5	2	1	3	1	2	6	1	2	3	26	5th
*Olea europaea* subsp. *cuspidata*	1	1	2	1	2	1	1	2	3	1	15	6th
*Embelia schimperi*	6	5	3	6	6	5	4	5	5	6	51	1st
*Tetradenia* sp.	2	6	4	2	3	3	2	4	1	4	31	4th
Enor												
*Leucas calostachys*	2	3	3	4	3	2	1	1	3	2	24	5th
*Melia azedarach*	1	1	2	3	1	1	2	3	1	6	21	6th
*Citrus medica*	5	6	4	6	4	3	4	6	5	1	44	3rd
*Cucumis dipsaceus*	4	4	6	5	5	4	3	5	6	4	46	2nd
*Embelia schimperi*	3	2	1	1	2	5	5	2	2	3	26	4th
*Fuerstia africana*	6	5	5	2	6	6	6	4	4	5	49	1st
Misha												
*Croton macrostachyus*	2	1	2	5	5	4	2	2	1	3	27	6th
*Melia azedarach*	1	2	4	6	6	1	1	4	4	1	30	5th
*Olea europaea* subsp. *cuspidata*	5	3	6	1	1	2	4	3	5	6	36	3rd
*Brucea antidysenterica*	4	5	3	2	3	6	5	5	6	5	44	1st
*Juniperus procera*	3	4	1	4	4	3	6	1	3	2	31	4th
*Clausena anisata*	6	6	5	3	2	5	3	6	2	4	42	2nd
Sekoru												
*Olea europaea* subsp. *cuspidata*	2	3	6	3	1	6	4	5	3	3	36	4th
*Embelia schimperi*	4	4	5	4	2	4	5	4	5	6	43	2nd
*Croton macrostachyus*	3	1	4	2	6	2	3	1	2	2	28	5th
*Solanecio mannii*	5	6	3	5	5	5	6	2	6	5	48	1st
*Hypericum quartinianum*	1	2	1	1	4	1	2	3	1	1	17	6th
*Protea gaguedi*	5	5	2	6	3	3	1	6	4	4	39	3rd

#### Preference ranking of factors threatening insecticidal and repellent plants

Informants from three study districts (Deri Saja Zuria, Enor and Misha) ranked agricultural expansion as the most serious threat to the insecticidal and repellent plants followed by overgrazing, and collection for firewood/charcoal and construction/furniture purposes; whereas, collection for construction/furniture purpose followed by firewood and charcoal use as the most threatening factors in Sekoru district (Table 7).

**Table 7:** Preference ranking of factors threatening insecticidal and repellent plants in Ghibe valley, southwestern Ethiopia, 2024

Scores given by key informants (A–K)												
Threatening factors	A	B	C	D	F	G	H	I	J	K	Total	Rank
Deri Saja Zuria												
Agricultural expansion	4	5	5	3	5	4	5	5	5	5	46	1st
Use for fire wood and charcoal making	3	3	2	4	4	5	3	4	3	3	34	3rd
Overgrazing	5	4	4	5	2	2	4	3	2	4	35	2nd
Use for construction and furniture making	2	2	3	1	3	5	1	2	4	2	25	4th
Medicinal use	1	1	1	2	1	3	2	1	1	1	14	5th
Enor												
Agricultural expansion	4	5	5	5	5	4	5	5	5	5	47	1st
Use for fire wood and charcoal making	2	2	3	2	1	2	1	2	1	2	18	4th
Overgrazing	5	4	4	4	3	3	4	3	4	4	38	2nd
Use for construction and furniture making	3	3	2	3	4	5	3	4	3	3	33	3rd
Medicinal use	1	1	1	1	2	1	2	1	2	1	13	5th
Misha												
Agricultural expansion	4	5	5	3	5	1	5	5	5	4	42	1st
Use for fire wood and charcoal making	3	3	2	4	4	4	2	4	2	3	31	3rd
Overgrazing	5	4	4	5	2	3	4	3	4	5	39	2nd
Use for construction and furniture making	2	2	3	2	3	5	3	2	3	2	27	4th
Medicinal use	1	1	1	1	1	2	1	1	1	1	11	5th
Sekoru												
Agricultural expansion	3	5	3	5	4	1	5	2	1	3	32	3rd
Fire wood and charcoal making	4	4	5	2	3	4	3	4	4	5	38	2nd
Overgrazing	2	1	2	1	2	3	2	3	2	2	20	4th
Use for construction and furniture making	5	3	4	4	5	5	4	5	5	4	44	1st
Medicinal use	1	2	1	3	1	2	1	1	3	1	16	5th

### Direct matrix ranking of selected multipurpose plants

Seven commonly used plant species with multipurpose role and eight use criteria in each study district were considered for direct matrix ranking exercises as presented in Table 8. *Olea europaea* subsp. *cuspidata* (Wall.G.Don) Cif. was ranked first most multipurpose plant followed by *Juniperus procera* Hochst. ex Endl. in Enor and Sekoru districts. *Olea europaea* subsp. *cuspidata* was ranked first most multipurpose plant followed by *Juniperus procera* in Deri Saja Zuria district. *Juniperus procera* was ranked first most multipurpose plant followed by *Eucalyptus globulus* Labill in Misha district. In Enor district, the local people harvested multipurpose plant species mainly for firewood, medicinal use and charcoal making which were ranked 1st, 2nd, 3rd, respectively. In both Deri Saja Zuria and Misha districts uses for firewood, fence and medicine which were ranked 1st, 2nd, 3rd, respectively, whereas in Sekoru district uses for medicinal, firewood and fence were ranked 1st, 2nd, 3rd, respectively.

**Table 8:** Direct matrix ranking of selected insecticidal and repellent plants by three herbalists in Ghibe valley, southwestern Ethiopia, 2024

Plant species	Use categories																																						
	Medicinal					Food				Forage				Construction						Fence					Fire wood					Charcoal				Furniture				Total	Rank
Enor																																							
*Olea europaea* subsp. *cuspidata*	2	1			4	0	0	0		5	4		5	5	5			4		3	4			2	5		5		5	4	5	3		4	5	5		85	1st
*Eucalyptus globulus*	4	3			3	0	0	0		1	0		1	5	5			5		5	5			4	4		5		5	5	5	3		2	3	2		75	3rd
*Croton macrostachyus*	5	4			5	0	0	0		2	1		1	2	3			4		1	3			3	4		4		3	5	4	4		4	2	5		67	4th
*Juniperus procera*	2	4			3	0	0	0		2	1		2	5	5			5		5	5			5	5		4		4	3	2	5		5	5	5		82	2nd
*Melia azedarach*	1	2			0	0	0	0		4	2		1	1	0			1		4	3			4	4		4		3	3	4	3		0	0	0		44	6th
*Justicia schimperiana*	4	5			4	0	0	0		1	1		2	0	0			0		1	2			1	4		3		3	1	2	2		0	0	0		36	7th
*Carica papaya*	5	5			5	5	5	5		4	4		3	0	0			0		1	2			1	2		2		2	1	1	0		0	0	0		53	5th
Total	71					15				48				55						64					80					68				47					
Rank	2nd					8th				6th				5th						4th					1st					3rd				7th					
Deri Saja Zuria																																							
*Olea europaea* subsp. *cuspidata*	3		2		4	0	0	0		5	4		4	5			5	5		5	5			5	5		5		5	4	5	5		5	5	4		95	1st
*Eucalyptus globulus*	4		3		3	0	0	0		2	2		1	5			5	5		5	4			4	5		5		4	4	4	5		2	3	2		77	4th
*Croton macrostachyus*	5		4		5	0	0	0		2	3		3	4			3	3		4	3			4	4		5		5	5	4	3		4	3	5		81	3rd
*Juniperus procera*	4		4		5	0	0	0		2	1		2	5			5	5		5	5			5	4		4		5	3	2	3		5	5	5		83	2nd
*Melia azedarach*	1		0		0	0	0	0		3	1		1	1			2	1		4	5			4	4		4		3	0	0	0		0	0	0		34	7th
*Premna schimperi*	1		3		0	0	0	0		1	1		2	0			0	0		3	2			4	4		5		4	1	3	2		0	0	0		36	6th
*Carica papaya*	5		3		5	5	5	5		5	2		4	0			0	0		1	2			1	4		2		2	1	1	0		0	0	0		53	5th
Total	64					15				53				59						80					88					55				48					
Rank	3rd					8th				6th				4th						2nd					1st					5th				7th					
Sekoru																																							
*Olea europaea* subsp. *cuspidata*	3			5	4	0	0	0		4		4	3	5		5			4	3		2		2	5			5	5	4	5		3	4	5	3		83	1st
*Eucalyptus globulus*	4			3	3	0	0	0		2		2	1	5		5			5	3		3		4	4			3	4	3	2		3	2	3	2		66	3rd
*Croton macrostachyus*	5			4	5	0	0	0		2		1	1	2		3			2	1		3		3	4			4	3	3	4		3	3	2	5		63	4th
*Juniperus procera*	4			4	3	0	0	0		2		1	2	5		5			5	5		5		5	3			4	4	2	2		3	5	5	5		79	2nd
*Carissa spinarum*	3			4	3	2	3	2		4		5	5	1		2			1	4		3		4	4			4	3	1	0		0	0	0	0		58	5th
*Premna schimperi*	4			5	2	0	0	0		1		1	2	0		0			0	1		2		1	4			3	3	1	2		2	0	0	0		34	7th
*Carica papaya*	5			4	5	5	5	5		4		4	3	0		0			0	1		2		1	2			2	2	1	1		0	0	0	0		52	6th
Total	82					22				54				55						58					64					45				44					
Rank	1st					8th				5th				4th						3rd					2nd					6th				7th					
Misha																																							
*Olea europaea* subsp. *cuspidata*	2			5	0	0	0		0	3	1		3	4		5			4	3		2	2		5	5			5	4	5		3	4	2		0	67	3rd
*Eucalyptus globulus*	4			3	3	0	0		0	1	0		1	5		5			5	4		5	4		5	5			5	3	4		4	2	1		2	71	2nd
*Croton macrostachyus*	5			4	5	0	0		0	2	1		0	2		3			2	1		2	3		4	5			3	5	4		2	4	2		2	61	4th
*Juniperus procera*	4			4	5	0	0		0	2	0		2	5		5			5	5		5	5		3	4			4	2	3		3	5	5		5	81	1st
*Melia azedarach*	1			0	3	0	0		0	1	0		3	1		0			0	4		3	4		4	4			5	1	3		0	0	0		0	37	6th
*Calpurnia aurea*	2			1	2	0	0		0	1	0		1	0		0			0	1		2	4		4	4			2	1	0		0	0	0		0	25	7th
*Carica papaya*	0			2	1	5	5		5	4	4		5	0		0			0	1		0	1		1	3			2	0	1		0	0	0		0	40	5th
Total	56					15				35				51						61					82					48				34					
Rank	3rd					8th				6th				4th						2nd					1st					5th				7th					

### The Jaccard’s similarity index in the use of insecticidal and repellent plants between the study districts

The Jaccard’s similarity index was calculated to compare similarity in the use of insecticidal and repellent plant between any four study districts (Table 9). The value of Jaccard’s similarity index ranged from 0.15 to 0.36. The highest similarity index was observed between the Deri Saja Zuria and Sekoru (0.36) while the greatest difference was observed between the Enor and Misha (0.15).

**Table 9:** Jaccard’s similarity index of study districts along the Ghibe valley, southwest Ethiopia, 2024

Districts	Deri Saja Zuria	Enor	Misha	Sekoru
Deri Saja Zuria	1	0.23	0.17	0.36
Enor	0.23	1	0.15	0.21
Misha	0.17	0.15	1	0.19
Sekoru	0.36	0.21	0.19	1

### Comparison of knowledge between informant groups

Through analysis of knowledge of informants, the traditional usage of insecticidal and insect repellent plants in relation to districts, age, gender, education and experience was determined (Table 10). Data using one-way ANOVA test showed that there was a significant difference in the mean numbers of reported plants between study districts (*p* = 0.00), of which higher number of plants were reported from Deri Saja Zuria district as compared to those from the other three study districts, and between age groups (*p* = 0.00), where a higher number of plants were reported by older informants of 60 years of age and above. The result of Duncan analysis method used for pairwise comparisons showed that the average number of reported plants by people of Misha district was significantly lower than that reported by the people of Deri Saja Zuria, Sekoru and Enor districts. Similarly, the average number of reported plants by the people of older age group (> 60 years old) was significantly higher than the people of middle and younger age groups.

**Table 10:** Relationship between average number of plants and informant groups in Ghibe valley, southwestern Ethiopia, 2024

Variable	Variable category	Number of informants	Average number of plants ± SD	*P*-value
Districts	Enor	78	2.73^**b**^ ± 1.47	0.00
	Misha	96	1.40^**a**^ ± 1.24	
	Deri Saja Zuria	98	3.72^**c**^ ± 2.84	
	Sekoru	89	2.89^**b**^ ± 2.10	
Gender	Male	253	2.97 ± 2.28	0.00
	Female	108	2.01 ± 1.85	
Age groups	22–39	113	1.82^**a**^ ± 1.69	0.00
	40 -60	137	2.53^**b**^ ± 1.80	
	> 60	111	3.75^**c**^ ± 2.66	
Experiences	Key	77	4.82 ± 2.61	0.00
	General	284	2.11 ± 1.67	
Education	Literate	279	2.68 ± 2.25	0.96
	No formal education	82	2.70 ± 2.05	

Note that average number of plants followed by different letters in the same column are statistically significant (Duncan analysis)

Analysis of data using t-test showed that there was a significant difference in the mean numbers of reported plants between gender (*p* = 0.00), where higher number of plants were reported by men informants than women informants, and between informants of differing level of experience (*p* = 0.00), where a higher number of plants were reported by key informants than by general informants. However, there was no significant difference in the mean numbers of insecticidal and insect repellent plants reported between informants of different education levels (literate and no formal education) (*p* = 0.96).

## Discussion

Communities residing in the Ghibe valley of southwest Ethiopia use herbal medication prescribed by practitioners of traditional medicine. The current study attempted to compile insecticidal and repellent plant species used in the study area where malaria is largely endemic in order to identify potential candidate plants that may be produced as insecticides and repellents principally utilized to combat malaria-transmitting mosquitoes. The findings of the current study showed that there is difference in the number and species of insecticidal and insect repellent plants among the four different ethnic groups, which is in line with other similar studies in Ethiopia and elsewhere in the world [21, 39]. This difference could be due to variations in the rate of malaria transmission; different nearby plants and different environments could lead to variations in the use of insecticidal and insect repellent plants among the study districts. In the districts of Deri Saja Zuria, Enor and Sekoru malaria transmission is considerable because of the suitability of habitats for mosquito’s larval development. Hence, comparatively a greater number of plant species were reported from these districts compared to Misha district where malaria transmission is less common. Most of the documented plant species were used to repel mosquitoes. In the current study areas, malaria is a major concern that could contribute to the widespread usage of plants to deter mosquitoes that transmit malaria. The above finding is supported by results of other prior studies carried out elsewhere in different parts of Ethiopia [24, 40].

When comparing the current study locations to other Ethiopian locations, relatively higher number of insecticidal and or insect repellent plant species were reported to control arthropod pests, such as mosquitoes, houseflies, lice, ticks and other crop and animal pests. A number of similar previous studies from Ethiopia such as in Raya-Azebo district, Northern Ethiopia [24], Jimma zone, southwest Ethiopia [29], Seweyna district, Bale zone, southeast, Ethiopia [41], Akaki district, Eastern Shewa [40], Western Hararghe zone, eastern Ethiopia [42], Kolla Temben district of Tigray, northern Ethiopia [39], Addis Zemen town of South Gonder zone [43] and Abitehnan district, West Gojjam [44] reported 35 plants used as both insecticidal and or repellent plants, 23 insect repellent plants, 19 mosquito repellent plants, 18 insecticidal, repellent and ethnoveterinary important plants, 13 insect repellent plants, 9 mosquito repellent plants, 8 mosquito repellent plants, and 8 insecticidal and or insect repellents plants, respectively. This implies that both an abundant of plants and higher historical uses for protection of themselves against insect bite by those plants with insecticidal and or repellent properties in the current study areas.

Comparatively higher number of insecticidal and or insect repellent plants were reported in the current study areas which belonged to the families Lamiaceae and Asteraceae, respectively, which may be due to their large species richness. One of the largest dicotyledonous families in Ethiopian flora, Lamiaceae has 170 species [45] and Asteraceae has 2500 species with majority of *Asteraceae* family members have therapeutic applications [46]. This may suggest that these families are more widely distributed geographically and that they contain an abundance of bioactive compounds that contribute significantly to the insecticidal and or repulsive properties [46–48].

Previous studies indicated that several of the plants such as *Allium sativum*, *Nicotiana tabacum*, *Justicia schimperiana, Melia azedarach, Olea europaea subsp. Cuspidata, Calpurnia aurea, Premna schimperi, Ruta chalepensis, Phytolacca dodecandra, Croton macrostachyus, Juniperus procera* and others reported in this study have been utilized as insecticides and/or repellents in Ethiopia and other parts of the world [21, 24, 40, 41].

The majority of insecticidal and repellent plants that have been documented in the current study were found in the wild, with a small number grown in home gardens. In consistent with the present results, studies from other parts of Ethiopia and elsewhere showed that the majority of locally used insecticidal and repellent plants were collected from the wild [25, 49–52]. In addition, the widespread use of herbaceous plants may also suggest that they are more widely available and abundant in the current study areas.

With the exception of a few insecticidal and insect repellent plants such as *Impatiens ethiopica*, *Olea europaea* subsp. *cuspidata*, and *Echinops kebericho*, the majority of species reported in the current study have low marketability. The limited marketability of these plants may be because of the fact that the majority of them are freely and openly harvested by users from their immediate environments or limited market demand [24, 53]. Prior research on ethnomedicinal plants revealed that healers traded medicinal plants at home rather than on the open market [54]. The idea is that the majority of traditional healers would rather keep their botanical remedies secret [45].

The findings of this study demonstrate that leaves were used more frequently for both as insecticidal and repellents than other plant parts. This may be due to the presence of higher concentration of active chemicals in the leaves. Several scholars reported the common use of leaves in Ethiopia and in the world [21, 24, 40, 41, 55]. Using leaves instead of other plant parts like roots, stems, and bark might be a more environmentally friendly option as leaves are available year-round. Most of the insecticidal and insect repellent plants were harvested and used while they were fresh, whereas very few were prepared from dried parts in line with previous similar studies [24, 40]. It is preferable to use fresh plant parts when seeking insecticides and insect repellents that have volatile oils as that may decrease with drying.

One of the most frequently used mode of applications was drinking the juice of plants to repel insects mainly for mosquitoes. These methods are comparable to the results of earlier studies carried out in other parts of the country [24, 41]. A plant juice that is taken orally may produce volatile substances through the mouth and skin that may deter mosquitoes [24]. House fumigation with smoke of burning plants was another most frequently used mode of applications both to kill and repel insects. Although no comprehensive investigation has been conducted to determine the exact method of action of smokes or their constituents, several earlier researchers have suggested the following potential theories for how plants work against insects. Burning plant parts may release volatile compounds that act as insecticides or repellents against insects [56, 57]. Additionally, smoke can hide the chemical substances that emerge from the host body, which is crucial for insects to locate their hosts, and it may disrupt the convection currents to the host location [41]. People in the current study areas also used certain plants to combat insect pests off their *E. ventricosum* plantation by growing the plants together and they protect their stored crops against storage insect pests by spraying the powder of plants and direct placing of fresh leaves that used as both insecticides and repellents same to the use reports of other similar earlier studies from Ethiopia and other parts of the world [58–60].

The most preferred plants used as insecticides and or insect repellents identified by preference ranking exercise of selected plants in the current study areas such as *Eucalyptus globulus, Croton macrostachyus*, *Calpurnia aurea*, *Juniperus procera, Melia azedarach*, *Echinops kebericho*, *Olea europaea* subsp. *cuspidata*, *Coleus abyssinicus,* and *Lippia abyssinica* may explain that they are most frequently used in the community.

Literature survey shows that of the total insecticidal and insect repellent plants recorded from the study area, 10 were assessed both for their insecticidal and insect repellent activities and phytochemistry, three for their activity insecticidal and insect repellent activities only and 11 for their phytochemistry only (Table 11), and these include some of the most frequently cited and most preferred insecticidal and insect repellent plant species recorded during the current study area for their use against malaria-transmitting mosquitoes and that have been reported in Ethiopia and elsewhere in the world with promising results [28, 29, 56, 61–64, 76, 81, 83] (Table 11). These include investigations conducted in Ethiopia on the larvicidal and adulticidal properties of essential oil derived from *Eucalyptus globulus* [63], the repellent activities from smoking leaves [76] and repellent activities of essential oil of *Echinops kebericho* againist *An. arabiensis* [83], larvicide activities of *Melia azedarach* seed powder [77] and repellent effects of seed oil [78], larvicidal activities of crude extract of *Calpurnia aurea* againist *An. arabiensis* and *An. stephensi* [79, 81], larvicide and adulticide activities of essential oil of *Lippia abyssinica* againist *An. arabiensis* [63], larvicide, repellent activities againist *An. arabiensis* and phytochemical screening of *Juniperus procera* [28, 29, 76], larvicide effect of crude extract of *Croton macrostachyus* [30] and larvicide effect of crude extract of *Olea europaea* subsp. *cuspidata* [80] and repellent activities of smoking leaves againist *An. arabiensis* [56, 62, 76]. All of these exemplifies that the traditional knowledge-based research could significantly aid in the development of new environmentally friendly insecticides or repellents. Nevertheless, less is known about the phytochemistry of these plant species that could offer protection from mosquitoes and or any other arthropod vectors.

**Table 11:** List of recorded plants from the current study area along with their results of previous studies on activities against malaria vectors and phytochemicals

Plant species reported from current study area	Parts extracted	Solvents used	Attempted bioassay test	Phytochemical compounds reported	References
*Ajuga integrifolia*	Leaves	Methanol, n-hexane	–	Alkaloids, flavonoids, phenol, glycosides, terpenoids, tannins, saponins, steroids	[93]
*Allium sativum*	Bulb	Aqueous, ethanol, methanol	Larvicide effect of extracts on different mosquitoes sp. and phytochemical analysis	Saponin, tannin, phenol, steroids, terpenes, anthraquinones, flavonoid, glycosides	[84]
*Artemisia annua*	Leaves	Ethanol, methanol	Larvicide effect of extracts on *An. arabiensis*	Artemisinin, scopoletin, arteannuin, arteannuic acid, scoparone, β-sitosterol	[79, 95]
*Calpurnia aurea*	Leaves	Ethanol, methanol	Larvicide effect of extracts on *An. arabiensis*	Alkaloids, saponin, tannin, phenol, flavonoid, terpenoids	[79, 87]
	Leaves	Aqueous, methanol	Larvicide effect on *An. stephensi*	Alkaloids, flavonoids, saponins, and tannins	[81, 98]
*Carica papaya*	Seed	Ethanol, n-hexane, ethanol, aqueous	–	alkaloids, saponin, phenol, flavonoids, glycoside, tannin, anthraquinone, steroid	[94]
*Citrus medica*	Peel	Ethyl acetate, ethanol	–	Tannin, phenol, flavonoid, coumarin, steroids, glycosides, carbohydrates	[92]
*Clausena anisata*	Leaves	Ethanol, methanol	Larvicide effect of extracts on *An. arabiensis*		[79]
*Coleus abyssinicus*	Leaves	n-Hexane, chloroform, methanol	–	Flavonoid, alkaloid, phenol, terpenoid, tannin	[96]
*Croton macrostachyus*	Leaves, Stem bark	Methanol	Larvicide effect of extracts on *An. arabiensis*	Cardiac glycosides, alkaloids, saponin, tannin, flavonoid	[30, 89, 96]
*Echinops kebericho*	Roots	Water	Repellent effect of essential oil on *An. arabiensis*	Terpenoids, flavonoid, glycosides	[83]
*Eucalyptus globulus*	Seed		Repellent effect of smoking on *An. arabiensis*		[76]
	Leaves, Seed	Water	Larvicide and adulticide effect of oil on *An. arabiensis*	Saponin, tannin, phenol, glycosides	[63, 88]
*Foeniculum vulgare*	Leaves	Water, methanol, chloroform	–	Alkaloids, tannins, saponins, terpenoids, flavonoids, steroid	[99]
*Juniperus procera*	Leaves		Repellent effect of and smoking *An. arabiensis*		[76]
	Leaves	Water	Larvicide effect of oil againist *An. arabiensis*		[28, 29]
*Justicia schimperiana*	Root	Methanol, chloroform	–	Alkaloids, phenols, tannins, saponins terpenoids, steroid, flavonoids, glycosides	[96]
*Lippia abyssinica*	Leaves	Water, methanol, ethyl acetate	Larvicide and adulticide effect of oil* An. arabiensis*	Alkaloid, phenolics, tannins, saponins flavonoids	[63, 92]
*Melia azedarach*	Seed		Larvicide effect of powders on *An. arabiensis*		[77]
	Seed Leaves	Water	Repellent effect of seed oil on *An. arabiensis*	Alkaloids, phenols, tannins, saponins terpenoids, steroid (from leaves)	[78, 97]
*Nigella sativa*	Seed	Petroleum ether, ethyl acetate, methanol	–	Alkaloids, saponin, tannin, steroids, flavonoid, terpenoids, glycosides	[92]
*Ocimum basilicum*	Leaves		Repellent effect of smoking aginist *An.*mosquitoes		[85]
*Ocimum gratissimum*	Leaves	Methanol, aqueous	–	Alkaloids, saponins, tannins, phlobatannin, steroids, terpenoid, flavonoid, glycosides	[92]
*Olea europaea* subsp. *cuspidata*	Leaves		Repellent effect of smoking on *An. arabiensis*		[56, 62, 76]
	Leaves	Petroleum ether, chloroform, acetone, ethanol	larvicide effect of extract on *An. arabiensis* and phytochemical analysis	Alkaloids, carbohydrates, glycosides, saponins, phenols, phytosterols, proteins, tannins	[80]
*Phytolacca dodecandra*	Seed, leaves	Water	Insecticide effect of extract on *A. arabiensis*	Saponins, tannins, flavonoids, terpenoids,	[82, 86]
	Berries, root	Water	Larvicide effect of extract on *A. arabiensis*	Genins, glycosides and phenolics	[64]
*Rhamnus prinoides*	Root	Methanol, water	–	Triterpene, saponins, tannins, phenols, glycosides, cardiac glycosides, resins	[92]
*Ruta chalepensis*	Fruit	Methanol	–	Phytosterols, phenols, tannins, alkaloids, flavonoids, saponins	[90, 92]
*Thymus schimperi*	Aerial part	Water	–	Carvacrol, thymol, terpineol, linalool, terpinene, limonene, octanol, transcaryophyllene	[91]

According to the informants in the current study areas, the main threats to insecticidal and insect repellant plants included uses for firewood/charcoal making, construction/furniture purposes, overgrazing, agricultural development and medicinal usage. This may be because of the growing population and demand for farmland, as well as the long history of timber production in the current study areas which is consistent with several previous research findings on the threats of traditional medicinal plants [65–70]. Traditional healers claim that because some of the reported insecticidal and repellent plants are so rare these days, it takes a long time and requires traveling long distances to find them. This result is in line with findings of earlier research on medicinal plants in different parts of Ethiopia [71–73].

Direct matrix ranking activities identified different multipurpose plant species that are the most preferred and extensively exploited by the local communities across all the study districts. Direct matrix ranking helps to evaluate the relative importance of plants to the local people and the extent of the prevailing threats. These plants were mostly collected for furniture, medicine, animal feed, charcoal, fencing, food, fuel, and construction. Because of this, they were the most threatened plants in the study area, and their sustainability depends on measures taken to conserve them.

The knowledge of locals on medicinal plants may be impacted by sociocultural factors or the proximity of ethnic groups to one another, as revealed by results of the analysis of the Jaccard's similarity index in the use of insecticidal and repellent plants between the study districts which is in line with other ethnomedicinal plant study in Ethiopia [68, 74]. In comparison to the other communities in the current study locations, this would suggest that the Oromo and Yem people have the largest share of traditional knowledge of plants used as insecticides and repellents.

Analysis of informants' knowledge revealed significant differences (p = 0.00) between study districts in the mean number of reported insecticidal and insect repellent plants. Cultural variations can have a significant role in knowledge differences between the ethnic groups. Certain culturally representative plant usage could be difficult to share because of their unique cultural identities [21]. Men have better experience with plant contact, which could explain the notable difference in traditional insecticidal and repellent plant knowledge between men and women informants. This finding is contrary to results of other similar earlier studies in northern parts of Ethiopia [24]. On average, older informants reported more plants, while younger informants reported less plants. This finding might point to a problem with the tranfer of knowledge from generation-to-generation regarding the use of insecticidal and insect repellant plants. According to similar earlier studies and other medicinal plant studies carried out elsewhere in Ethiopia, the lack of interest in learning and practicing of younger generations may be the cause of their poor level of awareness of plants with insecticidal and insect-repelling properties [24, 68]. Compared to the general informants, key informants mentioned a notably more insecticidal and repellent plants. This result is supported by a similar study conducted elsewhere in Ethiopia [68]. This is due to the fact that the key respondents were traditional herbalists who have extensive and empirical knowledge about the use of plants locally.

## Conclusions

The current study demonstrates that the local people in Enor, Deri Saja Zuria, Misha, and Sekoru districts use plants for protecting themselves against mosquitoes and other arthropod pests that transmit disease, destroy crops, and annoy people and domestic animals. This is evidenced by the use of 53 different plant species with insecticidal and repellent properties. Most of them were used as insect repellents, mostly for malaria-transmitting mosquitoes. The plants *Melia azedarach*, *Echinops kebericho*, *Olea europaea* subsp. *cuspidata*, *Coleus abyssinicus, Eucalyptus globulus, Croton macrostachyus*, *Calpurnia aurea*, *Lippia abyssinica, Juniperus procera, Phytolacca dodecandra* were found to be the most preferred plants as revealed by the highest frequency of citation and preference ranking scores in each study district. Therefore, among the most preferred plants future screening studies on the insecticidal and or insect repellent activities should give priority to *Coleus abyssinicus, Croton macrostachyus* and *Echinops kebericho* which have not yet been investigated in depth primarily against mosquitoes that transmit malaria in Ethiopia to validate their effectiveness in both under laboratory and field conditions.

## Data Availability

The datasets used and/or analyzed during the current study may be obtained from the corresponding author on reasonable request.

## Abbreviations

ANOVA: Analysis of variance
FC: Frequency of citation
IRS: Indoor residual insecticide spraying
JI: Jaccard’s similarity index
LLINs: Long-lasting insecticide-treated mosquito nets
SPSS: Statistical Package for the Social Sciences
